# Can We Improve Antifungal Susceptibility Testing?

**DOI:** 10.3389/fcimb.2021.720609

**Published:** 2021-09-10

**Authors:** Charlotte Durand, Danièle Maubon, Muriel Cornet, Yan Wang, Delphine Aldebert, Cécile Garnaud

**Affiliations:** ^1^TIMC, Univ Grenoble Alpes, CNRS, Grenoble INP, Grenoble, France; ^2^Parasitology-Mycology, CHU Grenoble Alpes, Grenoble, France; ^3^Inovotion SAS, Grenoble, France

**Keywords:** Antifungals susceptibility testing, MALDI-TOF, flow cytometry, molecular biology, computed imaging

## Abstract

Systemic antifungal agents are increasingly used for prevention or treatment of invasive fungal infections, whose prognosis remains poor. At the same time, emergence of resistant or even multi-resistant strains is of concern as the antifungal arsenal is limited. Antifungal susceptibility testing (AFST) is therefore of key importance for patient management and antifungal stewardship. Current AFST methods, including reference and commercial types, are based on growth inhibition in the presence of an antifungal, in liquid or solid media. They usually enable Minimal Inhibitory Concentrations (MIC) to be determined with direct clinical application. However, they are limited by a high turnaround time (TAT). Several innovative methods are currently under development to improve AFST. Techniques based on MALDI-TOF are promising with short TAT, but still need extensive clinical validation. Flow cytometry and computed imaging techniques detecting cellular responses to antifungal stress other than growth inhibition are also of interest. Finally, molecular detection of mutations associated with antifungal resistance is an intriguing alternative to standard AFST, already used in routine microbiology labs for detection of azole resistance in *Aspergillus* and even directly from samples. It is still restricted to known mutations. The development of Next Generation Sequencing (NGS) and whole-genome approaches may overcome this limitation in the near future. While promising approaches are under development, they are not perfect and the ideal AFST technique (user-friendly, reproducible, low-cost, fast and accurate) still needs to be set up routinely in clinical laboratories.

## Introduction

The number of invasive fungal infections (IFI) observed in the last decades has risen in line with the continually growing number of immunosuppressed patients (Brown et al., 2012). This has led to an increased use of systemic antifungal drugs recommended to treat these infections including echinocandins, polyenes, triazoles and flucytosine. In addition, the introduction of new, better-tolerated drugs, such as triazoles and echinocandins, has encouraged their use in prophylactic strategies leading to stronger selective pressure on fungi (Bailly et al., 2016). Antifungal resistance is an evolving threat compromising treatment efficiency (Lamoth et al., 2018). Of concern, some species, such as the emerging C*. auris*, are even multi-drug resistant. Antifungal susceptibility testing (AFST) is therefore of increasing importance for managing patients and adapting therapy (Eggimann et al., 2015; Ioannidis et al., 2020). It is mainly indicated (i) in patients with proven or suspected invasive fungal infection (in strains isolated from sterile body sites, or from non-sterile body sites in high-risk patients), (ii) when acquired resistance is suspected or (iii) in patients presenting with refractory, relapsing or breakthrough fungal infection (Cuenca-Estrella et al., 2012; Pappas et al., 2016; Ullmann et al., 2018).The development of antifungal stewardship programs is now highly encouraged as only one quarter of patients are receiving an adequate and early treatment. These programs aim to optimize treatment for each patient, by using the optimal agent, at the correct dosage and for the correct duration, consequently making the emergence of antifungal resistance less likely (Hamdy et al., 2017; Johnson et al., 2020). They also rely on close supervision of local epidemiology and antifungal resistance data obtained from the AFST of strains causing severe infections.

Several methods of AFST are currently used or under development (Berkow et al., 2020). For routine practice and optimal patient management, AFST techniques should be user-friendly, reproducible, low-cost, fast and accurate. This paper reviews and compares conventional and recently developed AFST methods for yeasts and molds.

## Conventional AFST Methods

Current AFST methods determine the susceptibility of a given fungal strain (in a pure culture) to a given antifungal drug. All of these methods are phenotypic, evaluating growth inhibition at defined concentrations of the drug in liquid or solid media. Most of them are quantitative as they measure the minimal inhibitory concentration (MIC), that is to say the minimal concentration of drug required to inhibit fungal growth (**Tables 1** and **2**). Comparison of AFST techniques often relies on categorical agreement (=percentage of MICs classified in the same interpretive category between techniques) or essential agreement (=percentage of MICs within +/- 1 or 2 two-fold dilutions of those of the reference method).

**Table 1:** Current and innovative AFST methods.

Method		Time-to-result^a^	User-friendly	Quick to perform	Mold analysis	Quantitative	Level of automation	Phenotypic/genomic	Cost	Comments
**Reference**	Broth microdilution CLSI / EUCAST	24-48h	No	No	Yes	Yes	Partially	Phenotypic	€€	Standardized Ranges for reference strains available as QC^g^ Used to define CBP or ECOFF Operator dependent Subject to trailing endpoint
	CLSI disk diffusion	24-48h	No	No	No	No	None	Phenotypic	€	Standardized Ranges for reference strains available as QC^g^
	EUCAST Agar assay for *Aspergillus fumigatus*	24-48h	No	No	Yes *(Af^b^)*	No	None	Phenotypic	€	Standardized Ranges for reference strains available as QC^g^ Screening method Commercialized as VIPcheck™
**Commercialized**	Sensititre™ YeastOne™	24h^c^	Yes	Yes	No	Yes	May be partially	Phenotypic	€€	Ranges for reference strains available as QC^g^ and reference strains supplied in Culti-Loops™ format Fixed antifungal panel Subject to trailing end point
	Vitek2	9-24h average 15.5h	Yes	Yes	No	Yes	Fully	Phenotypic	€€€	Low correlation with reference methods for rare species
	ATB Fungus 3	24-48h	Yes	Yes	No	No	Partially	Phenotypic	€	Operator dependent Subject to trailing endpoint Differences between automatic and visual reading
	Neo-Sensitabs™	24-48h	Yes	Yes	No	No	None	Phenotypic	€	Ranges for reference strains available as QC^g^ Low correlation with reference methods Subject to trailing endpoint
	VIPcheck™	24-48h	Yes	Yes	Yes *(Af^b^)*	No	None	Phenotypic	€	Screening method
	Etest^TM^ and Liofilchem^TM^ strips	24h^d^	Yes	Yes	Yes^e^	Yes	None	Phenotypic	€€	Ranges for reference strains available as QC^g^ Operator dependent Subject to trailing endpoint
	MycoGenie^TM^	/	Yes	Yes	Yes	No	Partially	Genomic	€€	Mutation detection directly from clinical samples Non exhaustive (known mutations only)
	AsperGenius^TM^	/	Yes	Yes	Yes	No	Partially	Genomic	€€	
	Fungiplex *Aspergillus* ^TM^	/	Yes	Yes	Yes	No	Partially	Genomic	€€	
**Innovative**	MALDI-TOF MS	3-15h	No	No	Yes^f^	Yes	Partially	Phenotypic	€€	Technically challenging Still need extensive clinical validation
	MBT-ASTRA	7h	No	No	No	Semi	Partially	Phenotypic	€€	Still need extensive clinical validation
	Flow cytometry	30min -1h	No	Yes	Yes	No	Partially	Phenotypic	€€€	Non-standardized protocols Toxicity of the dyes, background noise
	Computed imaging	8-16h	No	No	Yes	No	Partially	Phenotypic	€€€	Operator independent Not subject to trailing endpoint Still need extensive clinical validation
	PCR, qPCR, multiplex PCR using microspheres	2-5h	No	No	Yes	No	Partially	Genomic	€€-€€€	Mutation detection directly from clinical samples Non exhaustive (known mutations only)
	Sequencing, NGS, WGS	/	No	No	Yes	No	Partially	Genomic	€€-€€€	Exhaustive Expensive New mutations need to be linked to resistance

a from the time at which a pure culture of a strain is obtained.

b Aspergillus fumigatus.

c except for amphotericin B where 48h is needed.

d some C. glabrata strains may need 48h.

e not recommended for Zygomycetes.

f Aspergillus spp.

g Quality Control.

**Table 2:** Antifungal agents tested and mutations detected by commercial antifungal susceptibility testing assays.

		Azoles					Echinocandins			Polyenes	Pyrimidines
		FLC	ITC	VRC	PSC	ISA	CSF	MCF	ANF	AMB	5FC
**Phenotypic**	Sensititre™ YeastOne™	x	x	x	x		x	x	x	x	x
	Vitek2	x		x	x		x	x		x	x
	ATB Fungus 3	x	x	x						x	x
	Neo-Sensitabs™	x	x	x	x		x			x	x
	VIPcheck™		x	x	x						
	Etest™ and Liofilchem™ strips	x	x	x	x	x	x	x	x	x	x
**Genomic**	MycoGenie™	TR34/L98H (*CYP51A*)									
	AsperGenius™	TR34, L98H, Y121F, T289A (*CYP51A*)									
	Fungiplex *Aspergillus*™	TR34, TR46 (*CYP51A*)									

FLC, fluconazole; ITC, itraconazole; VRC, voriconazole; PSC, posaconazole; ISA, isavuconazole; CSF, caspofungin; MCF, micafungin; ANF, anidulafungin; AMB, Amphotericin B; 5FC, 5-fluorocytosine.

### Methods Performed in Liquid Media

#### Reference Methods From the Clinical and Laboratory Standards Institute and the European Committee for Antimicrobial Susceptibility Testing

**Figure 1:**
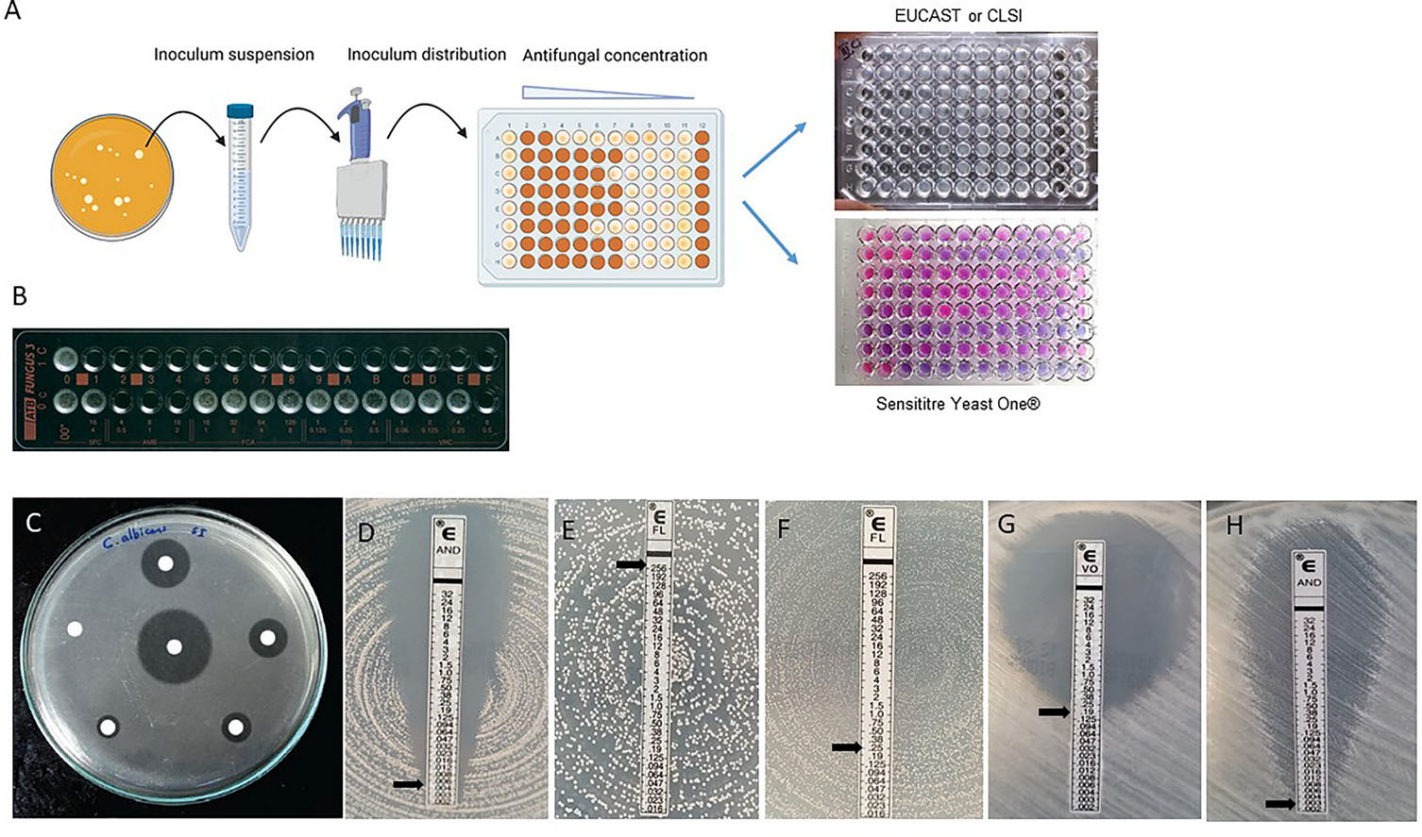
Conventional methods currently used for AFST. **(A, B)** In liquid medium. **(A)** Broth microdilution methods in 96-well microplate: EUCAST and CLSI references and Sensititre™ Yeast One™ **(B)** ATB Fungus 3. **(C–H)** In solid agar medium. **(C)** Disk diffusion method. **(D–H)** Gradient strips diffusion method. Black arrows indicate the MIC value. **(D)** *C albicans* isolate susceptible to anidulafungin; **(E)** *C albicans* isolate resistant to fluconazole; **(F)** *C albicans* isolate susceptible to fluconazole showing a strong trailing effect **(G)** *A fumigatus* isolate susceptible to voriconazole **(H)** *A fumigatus* isolate susceptible to anidulafungin.

Both CLSI and EUCAST reference methods are broth microdilutions (BMD) allowing the determination of the MIC. Briefly, a precalibrated amount of the strain to be tested is incubated in presence of 2-fold serial dilutions of the drug in the wells of a microtiter plate. MIC is determined as the lowest drug concentration inhibiting fungal growth to a preestablished threshold, compared to a no-drug control well (**Figure 1A**).

The CLSI committee (formerly the National Committee for Clinical Laboratory Standards, NCCLS) was the first to propose AFST standardization and quality control. The initial NCCLS protocol was a broth macrodilution in tubes, but the method rapidly evolved into a microdilution assay using a 96-wells microtiter plate (National Committee for Clinical Laboratory Standards., 1992). The current standard is the M27 4^th^ edition, which describes susceptibility testing of yeasts responsible for invasive fungal infections, including *Candida* spp. and *Cryptococcus* spp. (Clinical and Laboratory Standards Institute, 2017b). The EUCAST committee also established its own standard for AFST of medically important yeasts: the E.DEF 7.3 microdilution assay (Rodriguez-Tudela et al., 2008; Arendrup et al., 2012; https://www.eucast.org/fileadmin/src/media/PDFs/EUCAST_files/AFST/Files/EUCAST_E_Def_7.3.2_Yeast_testing_definitive_revised_2020.pdf). This EUCAST protocol notably differs from the CLSI standard by a higher glucose concentration of the RPMI growth medium, the type of microplates, a higher inoculum and a spectrophotometer *vs* a visual reading of the results (Berkow et al., 2020).

Comparison between the two reference methods in the five most common species of *Candida* spp. (*C. albicans*, *C. glabrata*, *C. parapsilosis*, *C. tropicalis* and *C. krusei*) showed comparable results for susceptibility testing of systemic antifungals. High categorical agreements of >90% were found for amphotericin B, anidulafungin, micafungin, fluconazole, and itraconazole for all species. High essential agreements (>90%) were also found for micafungin and fluconazole (Cuenca-Estrella et al., 2002).

Standards for testing the susceptibility of filamentous fungi are also available from the two committees. In the CLSI mold protocol, the inoculum size and incubation time are increased compared to those of the yeasts protocol (Clinical and Laboratory Standards Institute, 2017a). The EUCAST proposes the E.DEF 9.3.2 procedure, which mainly differs from the yeast protocol by visually determining the endpoints (https://www.eucast.org/fileadmin/src/media/PDFs/EUCAST_files/AFST/Files/EUCAST_E_Def_9.3.2_Mould_testing_definitive_revised_2020.pdf). MICs are determined for each antifungal agent, except for echinocandins for which the minimum effective concentration (MEC), defined as the lowest concentration associated with morphological changes (aberrant, short, hyphal segments compared to long, unbranched hyphae of the growth control) is measured.

In order to predict the susceptibility of the strain and thus the likely outcome of the treatment, MICs must be interpreted according to predefined thresholds. Specific clinical breakpoints (CBP), which differ from one species to another, are available for the two reference methods. They allow the isolate to be classified as susceptible (S) at standard dosing regimen, intermediate or susceptible dose dependent or susceptible increased exposure (I), or resistant (R) when there is a high likelihood of therapeutic failure even when exposure is increased. The “S” category means a high likelihood of treatment success. The “I” category indicates that the susceptibility of the isolate depends on the dosing regimen and that dosing regimens achieving higher drug exposure are necessary. The EUCAST has designated an additional category named Area of Technical Uncertainty (ATU), which corresponds to MIC values around the CBPs for which categorization is doubtful (Arendrup et al., 2020). CBPs are determined using pharmacokinetic and pharmacodynamic data of the drugs, MICs distributions of wild-type (WT) isolates and outcomes of patients in clinical trials. However, CBPs are not defined yet for some species-antifungal agent combinations because of insufficient data. In these cases, other thresholds only based on the MIC distributions can be used: the Epidemiological Cutoff Values (ECOFF or ECV according to EUCAST and CLSI, respectively). These thresholds allow the distinction of WT isolates (MIC ≤ ECOFF/ECV) from likely non-WT isolates (MIC > ECOFF/ECV) which may have acquired resistance mechanisms and therefore may not respond to therapy. Because data used to establish these breakpoints can be different (e.g., MIC obtained by EUCAST are usually lower than those obtained by CLSI), each committee provides its own CBP or ECOFF/ECV breakpoints (Espinel-Ingroff et al., 2005). Given the complexities of AFST, and when possible, medical mycologists should report both the MIC value and its interpretation, in order to guide physicians in the choice of the optimal antifungal therapy (Arendrup et al., 2020).

Although these protocols are standardized and used as references, they are time-consuming, require considerable expertise and so are difficult to perform in clinical laboratories in which many strains need to be tested every day. Visual reading of the results, as well as slight variations in the protocol (i.e., within the range of the recommended inoculum size) may expose to intra- and interlaboratory variability. Quality control procedures using reference strains that are detailed in the procedures are therefore of key importance. Thus, EUCAST and CLSI techniques are mostly used as gold standards for confirming or comparing results obtained with another technique, determining breakpoints or testing new antifungal compounds (Cuenca-Estrella et al., 2010).

#### Commercial Tests Currently Available for AFST

##### Sensititre™ YeastOne™

The Sensititre™ YeastOne™ assay (ThermoFisher Scientific, Waltham, MA USA) is a broth microdilution technique close to the reference methods described above, but which uses colorimetric detection to facilitate the reading (**Figure 1A**). The growth medium contains resazurin, an indicator of cell viability that turns from blue to pink when oxidized by viable fungi: MICs are determined after 24h of incubation as the lowest antifungal concentrations at which the wells remain blue (= no growth) and interpreted according to the CLSI breakpoints.

The system was first developed to assess *C. albicans* susceptibility to fluconazole (Pfaller et al., 1994). Agreement with the CLSI method after 24h of incubation was excellent (97%). Additional *Candida* species and *Cryptococcus neoformans* and drugs (amphotericin B, fluconazole, flucytosine, itraconazole and ketoconazole) were then evaluated. Again, a high overall agreement with reference methods, from 84 to 99%, was found after 24h of incubation, except for amphotericin B for which an incubation time of 48h was needed (Pfaller and Barry, 1994; Pfaller et al., 1998). More recent studies, including more species, also found good agreements with the CLSI reference BMD method for the majority of species-drug combinations (Espinel-Ingroff et al., 1999; Alexander et al., 2007; Pfaller et al., 2012).

Although not recommended, several studies investigated the performances of the Sensititre™ YeastOne™ assay for AFST of filamentous fungi. In *Aspergillus* spp., the assay performed well, with high overall agreements (≥92%) with the CLSI reference method for voriconazole, posaconazole and itraconazole (Wang et al., 2018): lower performances were found for amphotericin B (Guinea et al., 2006; Wang et al., 2018). The Sensititre™ YeastOne™ assay may also be an alternative technique for AFST of non-*Aspergillus* moulds (Halliday et al., 2016).

Currently, due to its high level of concordance with reference methods and the ease with which it can be performed and read, the system is widely used in clinical laboratories. Additionally, Thermofisher Scientific have developed several semi-automatic or automatic systems to inoculate and read the plates, thus improving accuracy and ease-of-use.

Of note, only pre-defined combinations of compounds are available for IVD use: YO2IVD (flucytosine, fluconazole, caspofungin, voriconazole, itraconazole); YO3IVD (same as YO2IVD plus micafungin); and YO10 (same as YO3IVD plus posaconazole, anidulafungin and amphotericin B).

##### Vitek 2

The Vitek 2 (bioMérieux, Marcy l’Etoile, France) is a fully automated system based on spectrophotometric detection for species identification and MIC determination. Basically, both a fungal suspension of the yeast to be tested and a card containing dried antifungal agents (amphotericin B, caspofungin, micafungin, fluconazole, voriconazole, posaconazole and flucytosine) at 4 to 6 different concentrations, are introduced in the system, in which inoculation, growth and reading are automatically assessed.

The Vitek 2 system provides fast results: an average of 15.5 h of incubation was shown to be sufficient for amphotericin B, flucytosine, fluconazole and voriconazole MICs testing (Cuenca-Estrella et al., 2010), while even shorter times (9h) were sufficient for echinocandins and posaconazole (Peterson et al., 2011). Its rapidity and objectivity make it suitable for use in clinical laboratories. In addition, excellent essential agreements (>95%) with the EUCAST and the CLSI methods were found for *Candida* spp. and flucytosine, amphotericin B, fluconazole, voriconazole, posaconazole, caspofungin and micafungin (Pfaller et al., 2007; Posteraro et al., 2009; Cuenca-Estrella et al., 2010; Peterson et al., 2011). Nevertheless, very major errors (isolates classified as resistant by the reference method and susceptible by the commercial technique) were detected (Cuenca-Estrella et al., 2010). Another comparison with the Etest^®^ technique (see below) also revealed discordant results between both techniques, i.e for *C. krusei* and flucytosine or *C. kefyr* and amphotericin B. In addition, fluconazole MICs for *C. tropicalis* were found higher by Vitek 2 than Etest^®^ (Alfouzan et al., 2017). Also, species such as *C. auris*, *C. haemulonii* and other related rare species were misidentified and their amphotericin B and caspofungin MIC values were over-estimated, leading to the recommendation of cautionary use for rare yeasts (Kathuria et al., 2015; Alfouzan et al., 2017).

##### ATB Fungus 3

ATB Fungus 3 (bioMérieux) is another commercially available technique using a dilution method for AFST of *Candida* spp. and *Cryptococcus* spp. yeasts (**Figure 1B**). It consists in a 32-wells strip containing increasing concentrations of amphotericin B, 5-flucytosine, fluconazole, itraconazole and voriconazole. After inoculation and incubation for 24 to 48h, growth is read visually or automatically with the mini Api instrument (bioMérieux). Overall essential agreement with the CLSI BMD reference method was excellent (99.1%) in *Candida* spp. with visual reading of the results, it was impaired when MICs were determined automatically, especially for azoles (73.4-80.7%), mainly because of trailing growth (Zhang et al., 2014). In addition, the lack of an echinocandin in the panel limits its use in routine practice.

### Tests Performed on Solid Media

#### Reference Methods From the CLSI and EUCAST

##### Disk Diffusion

Disk diffusion is one of the oldest and most common techniques for antimicrobial susceptibly testing. In brief, disks infused with a given concentration of an antifungal drug are applied on a calibrated inoculated agar plate before being incubated for 24h (**Figure 1C**). Interpretation is based on measuring the inhibition zone diameters. The CLSI has developed a standardized protocol (M44-A2) for yeast susceptibility testing using disk diffusion (Clinical and Laboratory Standards Institute, 2009). This method showed categorical agreements of 87% for fluconazole and 95.2% for voriconazole to the CLSI reference microdilution method by testing 3227 *Candida* spp. in 47 centers participating in the ARTEMIS program (Pfaller et al., 2009). This AFST assay does not provide MIC values but zone diameters. Interpretive criteria and breakpoints are available for caspofungin, micafungin, fluconazole and voriconazole. These breakpoints are provided for the five most encountered species: *C. albicans, C. glabrata, C. parapsilosis, C. tropicalis* and *C. krusei* with the exception of *C. parapsilosis* and the echinocandins (Espinel-Ingroff, 2007a; Brown and Traczewski, 2008; Arendrup et al., 2011). This restricted number of combinations of species and antifungals for which interpretative criteria are available is the main limitation of this AFST making this method inappropriate for rare species and other antifungals. However, the fluconazole and voriconazole diameter breakpoints were applied to the large panel of *Candida* species (31 different species) included in the ARTEMIS collection of clinical isolates (Pfaller et al., 2010a). Other limitation of the disk diffusion method is the low number of commercially available disks infused with antifungals. For yeasts, this method is standardized, reproducible, cheap, rapid (24h), easy to perform and interpret. Thus it is suitable for large surveillance studies such as the ARTEMIS one, and for the routine use in clinical laboratories specifically in low income countries (Pfaller et al., 2009; Pfaller et al., 2010a). The disk diffusion method was applied to pathogenic molds leading to the M51-A CLSI standardized protocol (Clinical and Laboratory Standards Institute, 2010). However, evaluation studies showed insufficient correlation to the microdilution method and no interpretative criteria are available to date (Espinel-Ingroff et al., 2007b; Ozkutuk et al., 2008).

##### Agar Assay to Detect Azole Resistance in Aspergillus fumigatus

A standardized agar assay, consisting of a four-well agar plate containing no drug (control well), itraconazole (4 µg/mL), voriconazole (2 µg/mL) or posaconazole (0.5 µg/mL) to be incubated for 24 to 48h, has been recently developed by EUCAST: E.DEF 10.1 (Guinea et al., 2019). Its objective is to screen and rapidly identify azole-resistant *A. fumigatus* strains, but it does not provide MICs.

#### Commercial Tests

##### Disks Diffusion: Neo-Sensitabs™

Commercial antifungal disks (Neo-Sensitabs; A/S Rosco Diagnostica, Taastrup, Denmark) are available for routine AFST in clinical laboratories. Susceptibility of *Candida* spp. to fluconazole using these disks has been assessed, and 100% of resistant strains were correctly classified (Sandven, 1999). However, it was not possible to differentiate susceptible and intermediate strains (Sandven, 1999; Vandenbossche et al., 2002). Comparison with the CLSI disk diffusion and microdilution reference methods showed very good correlation for fluconazole, itraconazole, voriconazole and caspofungin, but not for amphotericin B, even after 48h of incubation. The Neo-Senitabs method did not detect some isolates with high amphotericin B MICs (Espinel-Ingroff et al., 2007c). Correlation with the EUCAST microdilution method was excellent for amphotericin B, flucytosine, fluconazole, itraconazole and voriconazole and in this study Neo-Sensitabs identified amphotericin B resistant isolates, but misclassified > 5% of fluconazole resistant isolates as susceptible (Cuenca-Estrella et al., 2005). Another study tested the susceptibility of *Candida* spp. and *Cryptococcus neoformans* to amphotericin B, 5-fluorocytosine, fluconazole, itraconazole, ketoconazole and miconazole. The authors reported up to 38% of discrepancies with the CLSI reference method, all with the azoles and mostly with fluconazole and ketoconazole (Swinne et al., 1999). One study suggested potential application for filamentous fungi and itraconazole, voriconazole, caspofungin and posaconazole and found good correlations with the E-test method except for amphotericin B (Colosi et al., 2012). From these several incongruent results it remains difficult to reach a consensus about the clinical utility of the Neo-Sensitabs assay for AFST.

##### Agar Assay to Detect Azole Resistance in Aspergillus fumigatus: VIPcheck™

The VIPcheck™ (Mediaproducts BV; Groningen, Netherlands) is an easy-to-use four-well plate containing RPMI agar medium with no drug (control), itraconazole (4 µg/mL), voriconazole (2 µg/mL) or posaconazole (0.5 µg/mL), similar to the EUCAST reference assay. Briefly, each well is inoculated with *A. fumigatus* conidia and growth is assessed after 24 and 48h of incubation. In a single-center evaluation, after 48h of incubation, this assay allowed the isolation of azole-resistant strains with mean sensitivity and specificity of 98% and 93%, respectively (Buil et al., 2017). An updated version including isavuconazole would be of interest because this antifungal is now recommended as an alternative to voriconazole in the first line therapy of invasive aspergillosis (Tissot et al., 2017). Of note, the VIPcheck™ is a screening assay, and resistance must be confirmed by MIC testing.

##### Agar Assay Using Etest® and Liofilchem® Gradient Diffusion Strips

First developed by AB Biodisk (Solna, Sweden), then commercialized by bioMérieux (Craponne, France), Etest^®^ is one of the most widely used agar diffusion methods for AFST of both yeasts and molds (Bellanger et al., 2020). MIC test strips for AFST are also available from Liofilchem (Roseto degli Abruzzi, Italy).

Gradient diffusion strips are made of plastic (Etest^®^) or paper (Liofilchem^®^) and contain both a gradient of an antifungal agent and a concentration scale allowing the MIC to be read. They are applied on agar plates inoculated with a pre-established amount of the strain to be tested. After incubation for 24 to 48h, an ellipse can be seen around the strips, due to the inhibition of fungal growth resulting from the diffusion of the antifungal agent. The MIC is read at the point where the ellipse crosses the scale. Reading guides and training are necessary to determine MICs accurately, as different patterns of growth (i.e macrocolonies or microcolonies) can be seen on the agar plate depending on the tested strain and antifungal (**Figures 1D–H**). Most studies showed good essential agreements between MIC values provided by the Etest^®^ technique and the BMD reference techniques (Berkow et al., 2020). In addition, Etest^®^ ECOFF for *Candida* spp. and *Aspergillus fumigatus* were found to correlate with CLSI and EUCAST ECOFF/ECV (Salsé et al., 2019). Therefore, MIC values obtained with Etest^®^ may be interpreted according to CBP (or by default ECOFF/ECV) obtained from the reference methods.

Etest^®^ proved to be an excellent method for testing azoles (fluconazole, itraconazole, voriconazole and posaconazole), flucytosine and amphotericin B susceptibility in *Candida* spp. with >90% overall essential and categorical agreements with the CLSI method (Alexander et al., 2007). Similar results were found for echinocandins, except for *C. krusei* and caspofungin (Pfaller et al., 2010b). However, as for other AFST techniques, duration of incubation may influence these results (Pfaller et al., 2000; Bellanger et al., 2020).

Diffusion strips can also be reliably used for triazoles and amphotericin B susceptibility testing of molds. High (>90%) categorical or essential agreements with the broth microdilution (BMD) reference methods were found for AFST testing of *Aspergillus* spp., except for posaconazole (Lamoth and Alexander, 2015; Idelevich et al., 2018). Regarding zygomycetes, Lamoth and colleagues found amphotericin B and triazoles AFST by Etest^®^ appropriate, whereas others showed low overall agreement (75.1%) with the EUCAST reference technique for amphotericin B and posaconazole and therefore it is not recommended in clinical practice. (Caramalho et al., 2015; Lamoth and Alexander, 2015).

### Benefits and Limitations of Current AFST Methods

The main benefit of current methods is their direct clinical interpretation through the S/I/R classification that helps clinical decision making (Arendrup et al., 2020). However, despite excellent correlation between the two reference methods, CLSI and EUCAST, their differences, specifically in their breakpoints, lead to a certain level of complexity in MICs interpretation and comparison between laboratories. Another advantage is that, with the exception of the reference methods, they are easy-to-use and thus have been performed in many clinical laboratories for many years, resulting in operators becoming highly skillful.

However, one of the main limitations of the current AFST methods is their turnaround time (TAT) because they are all based on the evaluation of growth inhibition. As fungi are slow-growing organisms, the total TAT from positive culture of the clinical sample to the AFST results is 24 to 72h (generally 24 to 48h for AFST once a pure subculture of the fungus is available, which already takes approximatively 12 to 24h to obtain). The total TAT of disk diffusion and gradient strips methods may be shortened by the direct inoculation of clinical samples in which a mono-species culture is likely. It has notably been proposed for positive blood cultures yielding *Candida* spp. and has reduced TAT by avoiding the subculture step, but validation with a wide range of drug-species combinations is lacking, especially with new azoles and echinocandins. Good categorical agreements have been shown for disk diffusion and fluconazole and voriconazole and for the gradient strips and fluconazole, voriconazole, isavuconazole, and caspofungin. Despite poorer results for amphotericin B and posaconazole the clinical utility of this direct AFST from blood cultures remains promising (Tan and Peterson, 2005; Guinea et al., 2010; Jabeen et al., 2016; Escribano et al., 2017; Oz and Gokbolat, 2018).

Another main limitation is that these techniques are operator-dependent. Even when a standardized method is used, results can be influenced by slight changes in inoculum size, incubation temperature and time, or by the fitness of the tested strain (itself possibly linked to resistance) (Vale-Silva and Sanglard, 2015). Tools used for reading are critical and recommendations for a visual or spectrophotometer reading should be followed. The trailing growth, defined as partial growth inhibition over a wide concentration range exceeding the MIC, complicates the evaluation of growth inhibition, may lead to an overestimation of antifungal resistance and can alter reproducibility. This feature, related to antifungal tolerance, is mostly observed with the fungistatic azoles which incompletely inhibit the growth of some *Candida* spp. likely due to the activation of the stress response pathways (Delarze and Sanglard, 2015; Maubon and Morio, 2018). The same phenomenon is seen with echinocandins and some mold species (Maubon and Morio, 2018). It impairs both the spectrophotometer (EUCAST) and visual (CLSI) readings. Similarly, the color change using the Sensititre™ Yeast One™ may not be obvious and distinct. It also explains the differences between automatic and visual reading frequently observed with azoles using ATB fungus (Zhang et al., 2014). Using solid media such as Etest^®^, the trailing effect is illustrated by microcolonies growing inside the ellipse of inhibition (**Figure 1F**).

Additional problems with the current methods are their inaccuracy to detect amphotericin B resistance, the difficulty to interpret the paradoxical growth phenomenon that may be seen with echinocandins and yeasts (which is different from the trailing effect) and their unreliability for caspofungin testing due to the lack of stability of the molecule in the culture media (Martín-Mazuelos et al., 2003; Espinel-Ingroff et al., 2013; Shields et al., 2013; Pfaller et al., 2014a; Pfaller et al.,2014b).

## Innovative AFST Methods Already Developed or Under Development

### Tests Based on MALDI-TOF

Matrix-Assisted Laser Desorption/Ionization Time-of-Flight (MALDI-TOF) mass spectrometry (MS) consists in analyzing proteins of a sample after ionization and laser irradiation. Ionized particles are separated according to their mass-to-charge (m/z) ratio and detected in a TOF analyzer, resulting in a mass spectrum in which each detected peak corresponds to a protein/peptide (mostly ribosomal or a component of the cell wall). In the microbiological field, the resulting spectrum displays a specific pattern which when compared to previously established and annotated spectra from databases, allows identification of the species level. The technique was first used for identifying bacteria thanks to their specific spectral fingerprints (Claydon et al., 1996; Holland et al., 1996) and then for fungal identification (Li et al., 2000; Welham et al., 2000).

In 2009, Marinach *et al.* hypothesized that global protein composition of fungi would change after drug exposition, and that MALDI-TOF MS could be used to determine the susceptible or resistant phenotype of a strain (Marinach et al., 2009). The proof-of-concept was successfully achieved with *C. albicans* and varying concentrations of fluconazole over 15h of incubation. A minimal profile change concentration (MPCC), corresponding to the minimum concentration at which the spectrum profile changed, was determined. Comparison of *C. albicans* resistant strains MPCC values with MIC values determined by the CLSI reference method showed correlations of 94% and 100% within a range of 1 or 2 2-fold dilutions respectively.

The MPCC was then more easily established with the introduction of the composite correlation index (CCI). Indeed, instead of directly displaying MPCC values, this new method used a matrix of correlation between all the spectra obtained at all drug concentrations (De Carolis et al., 2012). MPCC therefore corresponded to the lowest concentration that offered a spectrum closer to the one obtained with the maximal concentration (higher CCI value) than to the one obtained with the minimal concentration. The high categorical agreement of 94% between MPCC and MIC values proved this method to be reliable. In order to interpret MPCC results, MPCC breakpoints were determined as the lowest drug concentrations at which all spectra of known susceptible strains were similar to maximal concentration spectra and all spectra of known resistant strains were similar to the spectra of the culture without drug (De Carolis et al., 2012). The technique was further simplified using only 3 drug concentrations (Vella et al., 2013). Authors were able to classify all the 51 susceptible and 10 out of the 11 resistant isolates, which showed the high reliability of the method for *C. albicans* and caspofungin. They also decreased the duration of the experiment: 3h were sufficient (Vella et al., 2013). Triazoles were also tested with *C. albicans*, *C. tropicalis* and *C. glabrata*. Reproducibility of the MALDI-TOF AFST technique ranged from 54.3% to 82.9% according to the species and the drug. Similarly, essential agreement between MALDI-TOF and the CLSI reference method varied between 54.1 (*C. tropicalis*/fluconazole) and 97.1% (*C.glabrata*/posaconazole).Both parameters were improved when a 5% tolerance was applied for CCI ratios (Saracli et al., 2015).

Another study showed high accuracy of MALDI-TOF using clinical isolates of *C. tropicalis* and fluconazole in 4h, suggesting its possible application in clinical laboratories for rapid detection of resistant strains (Paul et al., 2018). Susceptibility to echinocandins of *C. parapsilosis* complex was also assessed after 4h and excellent agreements with reference techniques were found: 95% for anidulafungin and caspofungin, and 100% for micafungin (Roberto et al., 2020). For *C. glabrata* and anidulafungin, agreement was 85% after 3h and increased to 97% after 12h, suggesting that incubation time is critical (Vella et al., 2017).

Concerning molds, MALDI-TOF AFST was used to determine the susceptibility profile to voriconazole of 20 *Aspergillus* spp. isolates. Complete correlation with *CYP51* lanosterol-14α-demethylase sequencing and reference methods was obtained after 30 hours of incubation (Gitman et al., 2017).

#### MBT ASTRA

MALDI-TOF BioTyper Antibiotic Susceptibility Test Rapid Assay (MBT ASTRA) was first used for bacteria (Sparbier et al., 2016) and then extended to fungi (Vatanshenassan et al., 2018). Although this test relies on a MALDI-TOF technology, it is quite different from the MPCC/CCI one because it is based on analyzing the intensity of the peaks and areas under curve (AUC) displayed by the spectrum that can be correlated to the growth. By comparing AUC, MBT ASTRA provides a semi-quantitative analysis of the strain growth inhibition. When MBT ASTRA for caspofungin was compared to the CLSI method, its sensitivity ranged from 94.2% for *C. glabrata* to 100% for *C. albicans*, and its specificity from 80% for *C. glabrata* to 100% for *C. albicans*. Including a 6h incubation period, the overall time-to-results was 7h (Vatanshenassan et al., 2018). MBT ASTRA was also successfully used to directly analyze *C. glabrata* positive blood cultures and anidulafungin with an incubation time of 6h (Vatanshenassan et al., 2019).

In a recent meta-analysis, MBT ASTRA showed higher sensitivity than the methods relying on spectral changes (96% vs. 85.3%), while the specificity was similar for both approaches (93.2% vs. 94.2%, respectively). Interestingly, the pooled sensitivity and specificity of the methods with TAT <8h were higher (91.4% and 96.1%, respectively) than those with a TAT >8h (86.3% and 87.5%, respectively). Thus, with overall sensitivity and specificity of 91% and 95%, respectively, the MALDI-TOF MS-based AFST has shown encouraging results and may be considered as a new method for the rapid detection of resistance in fungi (Knoll et al., 2021).

#### Benefits, Limitations, and Perspectives

Despite these promising results, especially the short TAT, AFST techniques based on MALDI-TOF are still challenging regarding sample preparation and consequently are time-consuming. Therefore, they would gain in interest if they were fully automated.

A future perspective is to determine the susceptibility of a strain by detecting specific peaks of either sensitive or resistant strains directly on the raw spectra, without any incubation with drugs, as previously carried out for bacteria (Manukumar and Umesha, 2017). Determining the susceptibility profile using this approach seems promising since the association between *C. glabrata* clusters determined by MALDI-TOF and fluconazole resistance has been proven to be significant (Dhieb et al., 2015). However, as some clusters comprised both susceptible and resistance strains, the accuracy appears insufficient for immediate clinical use.

Overall, these AFST based on MALDI-TOF still need an extensive clinical validation with a wide range of species and antifungal drugs.

### Tests Based on Flow Cytometry

Although the first flow cytometer was developed in 1968, adapted instruments for microbiology were first used in 1983 to study bacterial growth and metabolism and analyze the effects of antibiotics (Boye et al., 1983). Since then, flow cytometry has been extensively used with multiple applications, including AFST, for both yeasts and filamentous fungi, although the latter have not been widely tested.

#### Principle and Dyes

Antifungal susceptibility by flow cytometry is classically evaluated by detecting alterations in cell viability in the presence of a drug. This approach is preferred over counting cells, as it reduces the incubation time. Briefly, fungal cells are incubated in presence of different concentrations of the antifungal drugs (from 30 min to 7h according to the study), and dyes are added before viability analysis by flow cytometry. Different viability dyes have been evaluated such as propidium iodide (PI), 2-choro-4-[2,3-dihy-dro-3-methyl-[benzo-1,3-thiazol-2-yl]-methyl-idene]-1 phenylquinolinium iodide (FUN-1), acridine orange (AO) and ethidium bromide. Analysis was based either on multi-fluorochrome staining to mark live and dead cells, or on single-staining with a change in the fluorescence intensity. Pore *et al*. first proposed PI and rose Bengal to analyze cell viability in the context of AFST (Pore, 1990). Since these promising results, many studies have used PI but further results were less conclusive depending on the drug and the experimental protocols (Pore, 1991; Green et al., 1994; Ramani and Chaturvedi, 2000; Chaturvedi et al., 2004; Benaducci et al., 2015). Besides PI, the two most promising fluorescent probes in AFST are AO and FUN-1. They have been reported as good dyes for fluconazole, 5-fluorocytosine, voriconazole, itraconazole and caspofungin susceptibility testing, giving excellent correlation with microdilution methods (Pina-Vaz et al., 2001; Pina-Vaz et al., 2005). However, results were unsatisfactory for amphotericin B and the couple *C. krusei* – caspofungin (Vale-Silva et al., 2012).

#### Benefits, Limitations, and Perspectives

Despite the short time-to-results (30 min to 1h with FUN-1 and AO), flow cytometry for AFST is not routinely used in clinical microbiology laboratories. Standardized protocols are lacking. In addition, it requires technical expertise and most of the laboratories are not equipped with such instruments.

### Tests Based on Computed Imaging

#### Principle

In tests based on computed imaging, fungal cells are shortly incubated in presence of antifungals, in a plate or on a slide depending on the assay. A fluorescent dye is then added and a set of high-quality images is automatically acquired. Images are further processed using a dedicated software to detect each fungal cell or microcolony. The signal detected depends on the dye used in the assay.

#### Viability Detection

The Cellometer Vision, previously used for other eukaryotic cells, was first evaluated as an image-based cytometric method to detect viable yeasts (Berkes et al., 2012). Compared to flow cytometry, computed imaging has the advantage of the possible subtraction of background noise. Intra-macrophage viability of *Histoplasma capsulatum* cells treated with amphotericin B or itraconazole was assessed by AO and PI staining: good correlation was found between viability determined by the Cellometer Vision and colony counting, the reference method used in this study.

#### Microcolony Detection

Another approach was based on microcolony imaging using a porous aluminum oxide (PAO) support (Ingham et al., 2012). Basically, PAO strips inoculated with *Candida* spp. were placed on RPMI plates containing different concentrations of antifungal agents (amphotericin B, anidulafungin, caspofungin, voriconazole and itraconazole). After incubation from 3.5h for amphotericin B and the echinocandins to 7h for the triazoles, the strips were stained with Calcofluor White and FUN-1 and imaged by fluorescence microscopy to monitor microcolony areas and growth inhibition. PAO MICs were determined based on the decrease in the average microcolony areas. Comparison with MICs assessed by the EUCAST method showed an average of 86% correlation for all antifungal agents.

#### Chitin Detection

More recently, the SensiFONG computed-imaging assay investigated cell wall chitin content for AFST (Wang et al., 2019). This method is based on the cell wall stress responses triggered by antifungal agents. Indeed, it was previously shown in yeasts that cell wall chitin content increased after exposure to caspofungin, thus protecting cells against cell wall damages (Walker et al., 2010; Wang et al., 2019). Resistant cells, as they are not damaged by the antifungals, do not develop this compensatory response and so do not exhibit variation in chitin content.

In the SensiFONG assay, *Candida* spp. and *Aspergillus* spp. were cultured respectively for 6 and 16h in the absence and with increasing antifungal concentrations (fluconazole, voriconazole, isavuconazole, anidulafungin and micafungin). Images were acquired after Calcofluor white staining. Differentiation between resistant and susceptible strains was based on variation of fluorescence intensity as compared to the control without antifungal. This computed imaging assay showed high categorical agreements with the EUCAST method (Wang et al., 2019).

#### Benefits, Limitations, and Perspectives

Compared to commercial AFST assays, computed imaging has a short TAT. In addition, the SensiFONG assay, which is not based on growth inhibition, is not compromised by trailing growth. However, tests based on computed imaging still need improvements before being used routinely as AFST techniques. Indeed, as flow cytometry, they require technical expertise, no standardized protocols exist and clinical microbiology laboratories are not equipped with such instruments and softwares yet.

### Tests Based on Molecular Methods

Molecular detection of resistance relies on nucleic-acid based assays: PCR, hybridization, targeted sequencing or whole-genome sequencing (WGS). It consists in detecting genetic alterations known to be associated with antifungal resistance (Shields et al., 2012; Arastehfar et al., 2020). Acquired resistance in fungi is mostly due to mutations in genes encoding antifungal targets and transporters, as well as transcription factors involved in these products (Maubon et al., 2014; Morio et al., 2017).

#### Molecular Methods for Susceptibility Testing in *Candida* Yeasts

##### Echinocandin resistance

Resistance of *Candida* spp. to echinocandins is associated with amino-acid substitutions in the *FKS* genes which encode their target: the 1,3-β-D-glucan synthase (Katiyar et al., 2006; Perlin, 2007; Garcia-Effron et al., 2009). Mutations associated with antifungal resistance are diverse but grouped into small hotspot regions of about 10 amino acids each: HS1 and HS2. In addition, detection of such a *FKS* mutation was shown to be predictive of therapeutic failure (Shields et al., 2012; Arastehfar et al., 2020). Different multiplex molecular techniques were therefore developed for the detection of these mutations in resistant (non-WT) isolates. However, to date, no commercial techniques are available and very few could be performed directly from a clinical sample (Kordalewska et al., 2019).

For instance, a method using classical multiplex PCR proved to be rapid (<4h) and efficient in (i) detecting the most common Fks1 and Fks2 mutations associated with echinocandin resistance in *C. glabrata* and *C. albicans*, and (ii) classifying strains as susceptible (WT) or resistant (presence of a *FKS* mutation) when compared with AFST by the reference CLSI BMD method, except for strains harboring heterozygous mutations (Dudiuk et al., 2014; Dudiuk et al., 2015).

Similarly, qPCRs using allele-specific probes were combined in a multiplex assay and allowed most of the *FKS1* mutations to be detected (including heterozygous ones) in *C. albicans* resistant strains (Balashov et al., 2006). The same principle was applied to *C. glabrata* using melting curve analyses and dual assays for both *FKS1* and *FKS2* and allows the identification of the most frequently encountered substitutions in only 3h (Zhao et al., 2016b).

Another multiplex assay, based on microspheres, was developed to detect known and new *FKS* mutations in *C. glabrata* using the Luminex Magpix technology (Pham et al., 2014). Briefly, probes detecting specific single nucleotide polymorphisms (SNPs) or wild-type sequences were bound to microspheres. After hybridization with the target, emission of fluorescence was assessed by the Luminex instrument, thus allowing the identification of mutations. High-throughput screening of 1,290 isolates was carried out and allowed 16 isolates with mutations to be identified, confirmed by sequencing. The method proved to be rapid as *FKS* profiles of 95 isolates can be determined in 5h.

Alternative and more exhaustive techniques are based on sequencing. Sanger sequencing has been widely used to detect *FKS* mutations in *Candida* spp (Shields et al., 2012; Beyda et al., 2014). More recently, Next-Generation Sequencing (NGS) has been shown to be a suitable method for the detection of *FKS* mutations (Garnaud et al., 2015; Spettel et al., 2019).The development of NGS in the last decades has also made whole-genome sequencing (WGS) of *Candida* yeasts easier. WGS analysis of resistant isolates allowed the identification of new potential mechanisms of resistance to echinocandins, such as *CDC6* mutations in *C. glabrata* (Singh-Babak et al., 2012; Chew et al., 2019; Consortium OPATHY and Gabaldón, 2019). However, to date, no such sequencing technique is available for routine microbiological diagnosis.

##### Azole Resistance

Molecular detection of azole resistance in *Candida* spp. is more complex than for echinocandins. Indeed, resistance is often multi-factorial, driven by several distinct mechanisms (Jin et al., 2018). The most common mechanisms are mutations leading to up-regulation of drug efflux transporters as ATP binding cassette (CDR1, CDR2, MDR1) or Major facilitator superfamily (MFS), or up-regulation/mutation of the *ERG11* gene encoding the azoles target (Maubon et al., 2014; White et al., 2015). Molecular methods used for detecting azole resistance are therefore based on detecting point mutations or measuring gene expression levels by qPCR.

Among them, a study showed that *CDR1* expression level in *C. glabrata* strains could determine azole resistance, with 100% sensitivity and 95% specificity (Gygax et al., 2008). Frade et al. described a multiplex method for the simultaneous quantification of *CDR1*, *CDR2*, *ERG11* and *MDR1* gene expression levels in *C. albicans* (Frade et al., 2004). As for echinocandin resistance, qPCR and melting curve analysis allowed point mutations to be detected in *ERG11* in *Candida* spp. (Loeffler et al., 2000). Interestingly, a melting curve analysis was recently developed to detect both *FKS* and *ERG* mutations in *C. auris* within 2h (Hou et al., 2019). This short time-to-results and the ability to concomitantly detect resistance to multiple classes of antifungal drugs are promising because multi-drug resistant isolates of *C. auris* or *C. glabrata* are emerging. Even more than for echinocandin resistance for which mutations are grouped into small HS regions, and as shown in different studies, the use of NGS and genome-wide approaches would be of a great value in detecting resistance to azoles in *Candida* because mutations are distributed all along the sequences of several genes (Garnaud et al., 2015; Castanheira et al., 2017; Consortium OPATHY and Gabaldón, 2019; Spettel et al., 2019).

#### Molecular Methods for Susceptibility Testing in Molds

##### Azole Resistance

*Aspergillus* resistance to azoles is acquired by two main mechanisms: *in vivo* under prolonged azole treatment, usually resulting in mutations in the coding region of the *CYP51A* target gene; or, more frequently, *ex vivo* due to the use of fungicides in agriculture, harboring chemical structures close to those of the antifungals used in humans (Denning and Perlin, 2011; Berger et al., 2017). The latter typically results in tandem repeats (TR) of several bases in the *CYP51A* promoter region, along with SNPs in its coding region. Among the mutations linked to environmental selection, those most described are the TR34/L98H and the TR46/Y121F/T289A. Other single-amino acid substitutions associated with azole resistance are also well described: M220, G54, G138 or G448. A recent study showed that azole pressure *in vivo* could also result in mutations in the *CYP51A* promoter, challenging the dogma of the “easy distinction” between these two routes of resistance acquisition based on the type of mutations (Buil et al., 2019).

In-house methods using PCR and sequencing have been extensively evaluated to study azole resistance in *Aspergillus* spp. (Denning et al., 2011; Zhao et al., 2016a). Quantitative PCRs with molecular beacons were also widely used to detect resistance to itraconazole and other triazoles (voriconazole, posaconazole and ravuconazole) by targeting the codons 54, 98, 138 and 220 (Balashov et al., 2005; Garcia-Effron et al., 2008). Recently, a surveyor nuclease assay was also developed to rapidly detect *CYP51A* point mutations in resistant *A. fumigatus* isolates (Arai et al., 2020).

Interestingly, detection of mutations linked to azole resistance can be performed either on *Aspergillus* cultures or directly from clinical samples. The latter is promising as negative cultures are frequent in patients with invasive aspergillosis. Real-time PCR methods have been developed for that purpose, and for instance, a TR34/L98H mutation in *A. fumigatus* was found within a brain biopsy and sputum samples (van der Linden et al., 2010; Denning et al., 2011; Zhao et al., 2016a). Techniques based on pyrosequencing of *A. fumigatus* DNA were also developed to assess azole resistance (van der Linden et al., 2010; Arai et al., 2020), and were optimized recently to be used directly on clinical respiratory samples (Chong et al., 2015; Dannaoui et al., 2017).

Commercial PCR methods have made azole resistance detection in *A. fumigatus* easier. These methods detect the fungus through the amplification of a rRNA multicopy gene (*A. fumigatus* only or *Aspergillus* spp.), thus allowing a rapid diagnosis of aspergillosis independently of resistance detection. Most of them associate the detection of resistance mutations in the *CYP51A* gene. It should be noted that the *CYP51A* gene is not a multicopy gene and thus the detection of resistance mutations is less sensitive than the detection of the fungus itself. MycoGenie (Ademtech, Pessac, France) is a real-time PCR assay targeting the 28S rRNA gene and the TR34/L98H mutation. Its comparison with an in-house real-time PCR showed 100% concordance and very good specificities and sensitivities were found for respiratory and serum samples. The limit of detection was below one copy for the 28S-rRNA gene and six copies for the *CYP51A* gene harboring the TR34/L98H alterations (Dannaoui et al., 2017). The AsperGenius real-time PCR assay (PathoNostics, Maastricht, the Netherlands) similarly detects the mutations in *A. fumigatus*. The method proved to be reliable when testing clinical samples such as bronchoalveolar lavages or sera (Chong et al., 2015; White et al., 2015). However, a TR34/L98H mutation in a resistant strain from a sputum sample was detected neither by MycoGENIE nor AsperGenius assays (Guegan et al., 2018). Another commercial method is the Fungiplex Aspergillus Azole-R IVD PCR (Bruker Daltonik GmbH, Bremen, Germany), detecting TR34 and TR46 mutations in the *CYP51A* gene in *A. fumigatus*. As these commercial methods only detect a few known mutations, their interest is dependent on the frequency and the type of mutations found in given regions worldwide.

##### Echinocandin Resistance

Molecular detection of echinocandin resistance in *Aspergillus* spp. is less developed than detection of azole resistance, as until now, acquired echinocandin resistance in *A. fumigatus* has been reported in only one clinical isolate from a patient with aspergilloma treated by azole and polyene therapy and then micafungin (Jiménez-Ortigosa et al., 2017).

##### Benefits, Limitations, and Perspectives of Molecular Methods

Overall, molecular methods based on PCR that detect mutations in genes encoding antifungal targets and/or transporters, as well as in the corresponding transcription factors, are now well described and some of them are even commercially available. They provide rapid results and, in some instance, can be applied directly on clinical samples. However, their main limitation is that they detect only a limited number of mutations and only known mutations that have been linked to antifungal resistance. This shortcoming can be resolved by sequencing and WGS approaches, that may reveal undescribed mutations. However, these sequencing-based methods extend time-to-results. In addition, when new mutations are detected, the link with resistance remains to be demonstrated.

### The Added Value of Machine Learning

Machine learning allows the analysis of complex data and could be integrated in routine practice in microbiology labs in the near future. In 2020, Peiffer-Smadja *et al.* listed 97 machine-learning systems already developed for clinical microbiology applications (Peiffer-Smadja et al., 2020). Among them, such a system was used to validate fluconazole EUCAST breakpoints in 2009 (Cuesta et al., 2009). In this study, data of 258 episodes of candidiasis were investigated. Eucast MICs of the isolates, doses of the antifungal treatment/MICs and treatment outcomes were analyzed using 5 computational algorithms to validate the EUCAST fluconazole breakpoints. Very recently machine learning was used to detect fluconazole resistance from MALDI-TOF spectra in tolerant *C. albicans* isolates displaying trailing growth (Delavy et al., 2019). In this study, spectra were acquired after incubation of yeasts with 0, 16 or 256 µg/mL of fluconazole and in the presence or absence of cyclosporin A, an inhibitor of tolerance to azoles. Three different algorithms were applied on housekeeping peaks from each spectra (RandomForest, Logistic Regression and Linear Discriminant Analysis). The most accurate and robust pipeline consisted in treating the strains with cyclosporin during 3h, without fluconazole, and using a Linear Discriminant Analysis algorithm. Validation of the pipeline was then made using characterized strains, and resulted in 83% sensitivity, 89% specificity, and 86% accuracy. Combining MALDI-TOF analysis and machine learning therefore appears to be a promising approach for AFST, but requires further validation.

## Discussion: Are Innovative Methods Better?

Slow growth and the trailing effect are the main problems of current AFST methods that are dependent on growth inhibition evaluation (i.e. EUCAST and CLSI reference methods in liquid or solid media, commercially available methods such as Sensititre™, Vitek 2, ATB fungus, Neo-Sensitabs™, Etest^®^ and Liofilchem^®^). Therefore, AFST may be significantly improved by measuring another signal, alone or in combination with growth inhibition. Detecting other fungal responses to antifungal stress may be faster, more accurate and more objective, all these features being critical to improve AFST for patient management and reproducibility.

This is what has been proposed with the use of the MALDI-TOF to characterize the changes in protein spectrum profiles between susceptible and resistant strains, with the exception of the MBT-ASTRA which still relies on growth. Further developments and simplifications, including machine learning, led to promising results with a short TAT of 3-4 hours. In addition, good agreements with the reference methods were found for the main *Candida* species (*C. albicans*, *C. tropicalis*, *C. parapsilosis*). However, these AFST techniques based on MALDI-TOF still need an extensive clinical validation with a large panel of species and antifungal drugs, and would also certainly gain in interest with automation of the workflow.

Molecular methods for AFST provide results within few hours (except sequencing), are usually highly sensitive and some of them may be applied directly on clinical samples skipping the culture step. These are clear advantages, which could lead to an early and adequate antifungal treatment and improvement of patient management. However, they also present some limitations. First, there is not always a direct link between the genotype and the phenotype: the detection of a mutation is not always associated with resistance. On the contrary, resistance cannot be ruled out in the absence of detection of mutations, as more than one underlying mechanism may account for resistant phenotypes, and that most of the molecular AFST techniques developed to date only target known mutations (Knabl and Lass-Flörl, 2020). In addition molecular detection is qualitative and does not provide a MIC value. The current introduction of numerous new and fast molecular-based tests such as “syndromic panels plus resistance detection” is challenging (Ramanan et al., 2018). It may result in high-throughput solutions for direct molecular susceptibility testing of various human specimens that will need clinical validation.

One crucial question raised when reviewing all the AFST possibilities is the need for quantitative or only qualitative results. MIC values are mostly interpreted using CBP or ECOFF/ECV and thus categorized in qualitative results: S/I/R (mostly used) or WT/non-WT classes by medical mycologists or clinicians. So why and when do we need quantitative evaluation of susceptibility? The MIC value may be relevant in specific cases, when there is a wide I category (intermediate or susceptible, increased exposure) as for *C. glabrata* and fluconazole (from 0.002 mg/L to 16 mg/L) or a wide susceptible category as for *C. parapsilosis* and echinocandins (as an example from 0.0001 to 4 mg/L for anidulafungin). Indeed, a *C. glabrata* isolate showing a MIC for the fluconazole of 8 or 16 mg/L may not motivate the same treatment decision than a strain with a MIC of 2 mg/L or less. Similarly with isolates of *C. parapsilosis* showing a MIC for anidulafungin of 0.002 mg/L and 2 mg/L. Also, for epidemiological study and surveillance, MICs are much more accurate for highlighting slight but significant modifications, and can be correlated to antifungal prescription in different statistical models, therefore contributing to antifungal stewardship (Fournier et al., 2011; Bailly et al., 2016).

To date, with the exception of flow cytometry and molecular methods, only one strain in a pure culture can be tested. Although rare (but possibly underdiagnosed), mixed populations and heterogeneity (i.e. subpopulations) cannot be easily detected (Gülmez et al., 2020). As subpopulations exhibiting different susceptibility patterns may be further selected and become predominant or persistent, their detection is of importance to adapt patient management and stop selective pressure when possible. The mixed populations constitute also a shortcoming to perform AFST directly from clinical samples.

## Conclusion

All current and innovative methods described above have some advantages and limitations, and none is perfect. The ideal AFST method needs to be fast, ideally less than 8h, to give results in a single working shift, so that the treatment may be adapted during the day. In addition, it needs to be culture independent, quantitative, functional in mixed populations and directly on clinical samples, low-cost and user-friendly. With the exception of very few molecular ones which are already on the market, all innovative techniques are still under investigation and need further development to achieve these goals. In addition, these techniques need to be fully automated and integrated in the entirely automated workflow now implemented in the clinical microbiology laboratories.

## Author Contributions

All authors listed have made a substantial, direct, and intellectual contribution to the work, and approved it for publication.

## Conflict of Interest

The authors declare that the research was conducted in the absence of any commercial or financial relationships that could be construed as a potential conflict of interest.

## Publisher’s Note

All claims expressed in this article are solely those of the authors and do not necessarily represent those of their affiliated organizations, or those of the publisher, the editors and the reviewers. Any product that may be evaluated in this article, or claim that may be made by its manufacturer, is not guaranteed or endorsed by the publisher.
